# Circulating TGF-*β*1 and VEGF and risk of cancer among liver transplant recipients

**DOI:** 10.1002/cam4.455

**Published:** 2015-04-27

**Authors:** Eric A Engels, Linda Jennings, Troy J Kemp, Anil K Chaturvedi, Ligia A Pinto, Ruth M Pfeiffer, James F Trotter, Michelle Acker, Nicholas Onaca, Goran B Klintmalm

**Affiliations:** 1 Division of Cancer Epidemiology and Genetics, National Cancer InstituteBethesda, Maryland; 2 Baylor Simmons Transplant Institute, Baylor University Medical CenterDallas, Texas; 3 HPV Immunology Laboratory, Leidos Biomedical Research Inc., Frederick National Laboratory for Cancer ResearchFrederick, Maryland

**Keywords:** Cancer, platelet, solid organ transplantation, transforming growth factor *β*, vascular endothelial growth factor

## Abstract

Transplant recipients have elevated cancer risk, perhaps partly due to direct carcinogenic effects of immunosuppressive medications. Experimental evidence indicates that calcineurin inhibitors given to transplant recipients increase cellular expression of transforming growth factor *β*1 (TGF-*β*1) and vascular endothelial growth factor (VEGF), which could promote cancer. To assess the potential role of these pathways in the transplantation setting, we conducted a case–control study nested in a cohort of liver recipients. Cases had nonmelanoma skin cancer (*N *= 84), cancer of the lung (*N *= 29), kidney (*N *= 20), or colorectum (*N *= 17), or melanoma (*N *= 3). We selected *N *= 463 recipients without cancer as controls. TGF-*β*1 and VEGF levels were measured in sera obtained, on average, approximately 3 years before case diagnosis/control selection. We also measured platelet factor 4 (PF4), a marker of ex vivo platelet degranulation, because TGF-*β*1 and VEGF can be released from platelets, and we developed a statistical model to isolate the platelet-derived fraction from the remaining circulating component. Compared with controls, lung cancer cases had higher levels of TGF-*β*1 (median 22.8 vs. 19.4 ng/mL, *P *= 0.02) and VEGF (277 vs. 186 pg/mL, *P* = 0.02). However, lung cancer cases also had higher platelet counts (*P* = 0.08) and PF4 levels (*P* = 0.02), while residual serum levels of TGF-*β*1 and VEGF, after accounting for PF4, were unassociated with lung cancer (*P* = 0.40 and *P* = 0.15, respectively). Associations were not seen for other cancers. In conclusion, TGF-*β*1 and VEGF levels were increased in association with lung cancer among transplant recipients, which may be explained by increased platelet counts and platelet degranulation in lung cancer cases.

## Introduction

Solid organ transplantation provides life-saving therapy for patients with end-stage organ disease. Cancer risk is elevated in transplant recipients 1, largely due to loss of immune control of oncogenic viruses arising from immunosuppressive medications administered to prevent organ rejection. Importantly, these medications may vary in their effects on cancer risk. Some epidemiological research suggests that calcineurin inhibitors (CNIs), an immunosuppressive medication class widely used in transplantation, may especially increase cancer risk 2,3. In comparison, sirolimus (a non-CNI medication that inhibits the mTOR protein) is associated with lower cancer risk 4,5.

Notably, not all the cancers that are increased in transplant recipients are caused by viruses 1, and the medications used in transplantation may affect cancer risk independently of the immunosuppression. In particular, experimental studies demonstrate effects of CNIs in mouse strains lacking an adaptive immune system, so their findings cannot be attributed to CNI suppression of T-cell immunity. Specifically, Hojo et al. found that administration of cyclosporine (a CNI) to SCID-beige mice dramatically increased the metastatic spread of tumor cells, and that this tumor-promoting effect was blocked by coadministration of antibodies to transforming growth factor-*β*1 (TGF-*β*1) 6. Additionally, Basu et al. demonstrated that cyclosporine upregulates vascular endothelial growth factor (VEGF) expression in cultured renal carcinoma cells 7. When these cells were injected into nude mice, administration of cyclosporine increased tumor growth. Thus, these studies point to mechanisms involving TGF-*β*1 and VEGF independent of immunosuppression.

TGF-*β*1 and VEGF play a role in development, growth, and metastasis of cancer. At early stages of carcinogenesis, TGF-*β*1 inhibits cellular proliferation, but cancer cells themselves later secrete TGF-*β*1, facilitating invasion, angiogenesis, and metastasis 8. VEGF acts as an important tumor angiogenesis signal 9. Numerous studies document elevated levels of TGF-*β*1 and VEGF in plasma or serum from cancer patients, including patients with melanoma and colorectal, kidney, and lung cancers 10–18. Importantly, however, circulating platelets store TGF-*β*1 and VEGF, and platelets release these proteins when a serum sample is created through clotting ex vivo, which can greatly increase serum levels 19–21. This methodologic issue is likely underappreciated, as most prior studies of cancer patients have not accounted for platelet release of TGF-*β*1 and VEGF in assessing total serum levels. The circulating platelet count can be used as an indirect measure of how much TGF-*β*1 and VEGF might be released by platelet degranulation. More directly, one can measure serum levels of platelet factor 4 (PF4), which is released by platelets when they degranulate 20.

Although laboratory studies suggest a role for TGF-*β*1 and VEGF in mediating carcinogenic effects of CNIs, no epidemiologic study has assessed TGF-*β*1 and VEGF in relation to cancer in solid organ transplant recipients. We, therefore, conducted a case–control study of cancer among liver recipients, many of whom were treated with CNIs to prevent graft rejection. We measured TGF-*β*1 and VEGF in serum specimens from cancer cases, obtained before cancer diagnosis, and at a comparable timepoint in recipients without cancer. Importantly, we also assessed the degree to which serum levels of TGF-*β*1 and VEGF were related to degranulation of platelet stores. Our approach and findings have relevance for other researchers evaluating the role of TGF-*β*1 and VEGF in cancer.

## Methods

### Study subjects

Beginning in 1985, all liver recipients at Baylor University Medical Center (Dallas, TX) have participated in a cohort study of transplant outcomes. Clinical data are collected at planned intervals and clinical events (e.g., acute rejection, hospitalization, death). Immunosuppressive medication use is ascertained at clinic visits. Information on smoking is not collected. Serum samples (stored at −80°C) are obtained at 0, 0.25, 0.5, 1, 2, 5, 10, and 15 years posttransplant. Participants provided informed written consent, and this study was approved by the Baylor institutional review board.

Cancers are identified through patient reports at follow-up visits and clinical record review. To focus on direct effects of CNIs, rather than those mediated by immunosuppression, we assessed cancers that are increased in transplant recipients, but are not known to be caused by viruses 1. We, therefore, included liver recipients diagnosed with nonmelanoma skin cancer (NMSC), lung cancer, kidney cancer, colorectal cancer, or melanoma. We excluded recipients who had hepatocellular carcinoma as the reason for transplant or detected in the explanted liver. We required that each case have a serum sample at least 3 months after transplant and at least 6 months before cancer diagnosis. We then selected the most recent serum sample within this interval.

For each cancer case, we selected up to 3 controls matched according to sex, race (white vs. non-white), age at transplantation (3 intervals), and time since transplantation (10 intervals). As for cases, we excluded recipients who had had hepatocellular carcinoma. Controls could develop one of the cancers of interest in intervals after their selection, and could be selected more than once in different intervals. We selected serum samples for controls at timepoints to match the cases as closely as possible. Finally, because 90% of cases and 93% of matched controls were receiving a CNI (either cyclosporine or tacrolimus) at the time of blood draw, we supplemented the controls with 22 recipients on sirolimus.

### Laboratory measurements

Serum levels of TGF-*β*1 were tested using a planar enzyme immunoassay (dilution 1:100; R&D Systems, Minneapolis, MN), and serum levels of VEGF were assessed on a Luminex bead-based platform (dilution 1:4; Bio-Rad Laboratories, Hercules, CA). To assess platelet degranulation, we measured serum levels PF4 using a Luminex bead-based platform (dilution 1:10,000; EMD Millipore, Billerica, MA) 20. Samples were assayed in duplicate and averaged to calculate concentrations.

### Statistical analyses

TGF-*β*1, VEGF, and PF4 levels were normalized using the square root (sqrt) transformation. We considered that total serum levels of TGF-*β*1 and VEGF reflect both the amount circulating in plasma in vivo and the amount released by platelet degranulation ex vivo (reflected by serum PF4 levels). These separate components can be estimated using linear regression. Specifically, using serum levels measured in controls, we fitted linear regression models of the form:

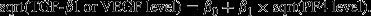


Based on these estimates of *β*_0_ and *β*_1,_ we calculated for each subject:

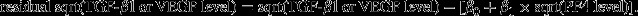


Because serum PF4 captures the component of total TGF-*β*1 or VEGF related to platelet degranulation, the above-calculated residuals estimate the in vivo plasma component.

We then used linear regression to assess associations of case–control status with total serum TGF-*β*1 or VEGF (sqrt-transformed), with PF4 levels (which captured the component related to platelet degranulation, sqrt-transformed), and with residuals (the component reflecting plasma levels). These analyses compared cases overall or for each type of cancer, with the entire group of controls. In additional models, we adjusted case–control comparisons for sex, age at transplantation, and sirolimus use, and we conducted an analysis for NMSC restricted to whites. Finally, we used linear regression to compare lung cancer cases to controls with respect to platelet counts (sqrt-transformed) and total serum levels of TGF or VEGF per platelet (log-transformed).

## Results

Cases (*N *= 153) and matched controls (*N *= 441) were appropriately similar (Table1). Among cases, NMSC was the most common cancer (*N *= 84), followed by lung cancer (*N *= 29), kidney cancer (*N *= 20), colorectal cancer (*N *= 17), and melanoma (*N *= 3). Cancers were diagnosed on average 7.0 years after transplantation, and sera were obtained on average 3.0 years before cancer diagnosis. We included 22 additional controls receiving sirolimus (*N *= 60 total controls on sirolimus).

**Table 1:** Liver recipients selected as cancer cases and controls

Characteristic	Cancer cases (*N* = 153)	Matched controls (*N* = 441)
Male sex, *N* (%)	101 (66)	287 (65)
White race, *N* (%)	143 (94)	411 (93)
Age at transplant in years, mean (SD)	51 (9.3)	51 (9.4)
Calendar year of transplant, mean (SD)	1994 (5.0)	1996 (5.2)
Cancer diagnosis, *N* (%)		
NMSC	84 (55)	
Lung cancer	29 (19)	
Kidney cancer	20 (13)	
Colorectal cancer	17 (11)	
Melanoma	3 (2)	
Years from transplant to selection, mean (SD)	7.0 (4.6)	6.6 (3.8)
Years from transplant to sample draw date, mean (SD)	4.0 (3.5)	3.6 (3.0)
Years from sample draw date to selection, mean (SD)	3.0 (3.0)	2.9 (2.5)
Calcineurin inhibitor on sample draw date, *N* (%)	138 (90)	410 (93)
Sirolimus use on sample draw date, *N* (%)	6 (4)	38 (9)

NMSC, nonmelanoma skin cancer; SD, standard deviation.

**Figure 1:**
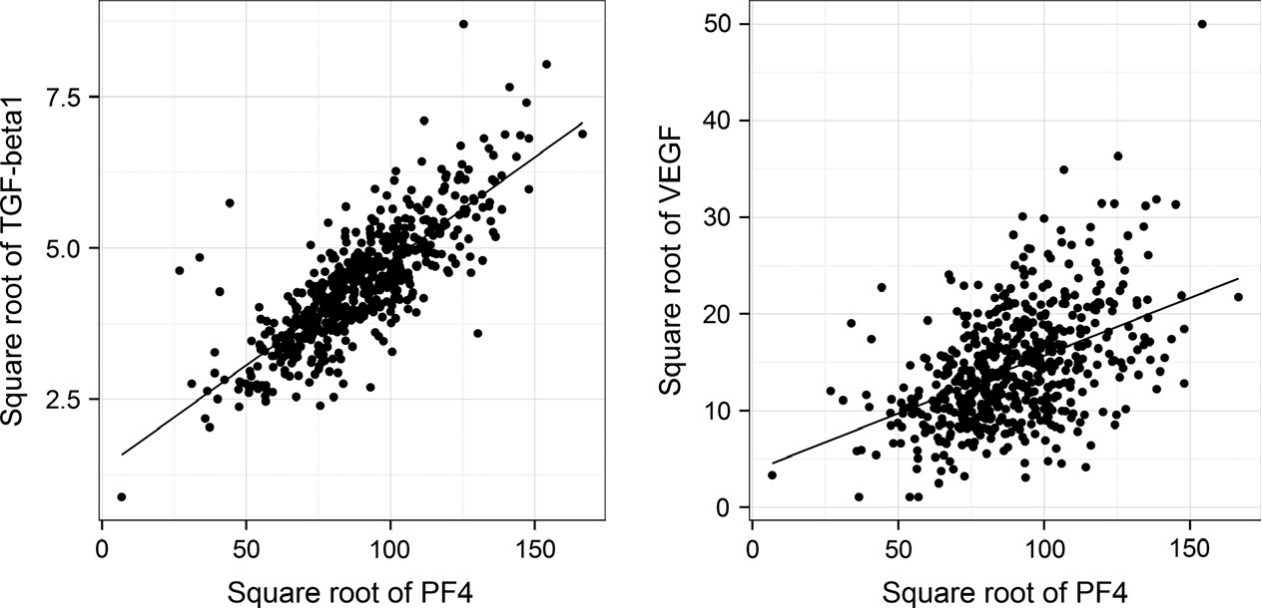
Relationships of total serum TGF-*β*1 (left panel) and total serum VEGF (right panel) with total serum PF4 levels. Levels are square-root transformed (TGF-*β*1 and PF4 levels are in ng/mL, and VEGF levels are in pg/mL). Results are shown for all subjects, along with the linear regression line derived from values among control subjects.

Serum TGF-*β*1 and VEGF levels were correlated with PF4 levels (*R *= 0.80, *P* < 0.0001, and *R *= 0.45, *P* < 0.0001, respectively, among all subjects combined; Fig.1). Figure1 also presents regression lines fitted among control subjects, showing the components of TGF-*β*1 and VEGF associated with PF4 levels and, thus, platelet degranulation. Serum TGF-*β*1 and VEGF were also correlated with platelet counts (*R *= 0.73, *P* < 0.0001, and *R *= 0.48, *P* < 0.0001, respectively).

Serum TGF-*β*1 and VEGF levels did not differ significantly between all cancer cases combined and controls (Table2). Likewise, PF4 levels and residual serum levels of TGF-*β*1 and VEGF did not differ overall (Table2).

**Table 2:** Associations of serum TGF-*β*1 and VEGF with case–control status

	Total serum level, median (IQR)		*P*-value for total serum level		*P*-value for PF4 level (reflecting platelet degranulation)	*P*-value for residual serum level (reflecting plasma level)	
Group	TGF-*β*1	VEGF	TGF-*β*1	VEGF		TGF-*β*1	VEGF
Controls	19.4 (14.2–24.5)	186 (107–315)	–	–	–	–	–
Cases overall	18.9 (14.2–24.4)	206 (107–303)	0.63	0.23	0.19	0.34	0.48
Cases by cancer type							
NMSC	19.2 (14.0–24.3)	190 (107–353)	0.97	0.54	0.70	0.65	0.62
Lung cancer	22.8 (16.8–29.6)	277 (112–303)	**0.02**	**0.02**	**0.02**	0.40	0.15
Kidney cancer	19.3 (14.6–21.7)	188 (84–258)	0.53	0.32	0.38	**0.02**	0.11
Colorectal cancer	14.8 (13.1–19.1)	201 (118–253)	0.29	0.93	0.49	0.40	0.65
Melanoma	17.2 (15.6–33.7)	457 (61–821)	0.66	0.14	0.59	0.98	0.16

Units are ng/mL for TGF-*β*1 and pg/mL for VEGF measurements. Bold results are statistically significant with *P* < 0.05. IQR, interquartile range; NMSC, nonmelanoma skin cancer; PF4, platelet factor 4; TGF-*β*1, transforming growth factor *β*-1; VEGF, vascular endothelial growth factor.

When we examined each cancer separately, total serum levels were significantly higher in lung cancer cases (TGF-*β*1: median 22.8 ng/mL, vs. 19.4 in controls; VEGF: median 277 pg/mL, vs. 186 in controls; Table2). Sera were obtained a mean of 6.8 years before lung cancer diagnosis. These differences in TGF-*β*1 and VEGF were due to greater platelet degranulation, since PF4 levels were higher in lung cancer cases than controls (*P* = 0.02), and residual serum levels did not differ between lung cancer cases and controls (*P* = 0.40 for TGF-*β*1, *P* = 0.15 for VEGF; Table2). Furthermore, platelet counts tended to be higher in lung cancer cases than controls (mean level 210,000 vs. 179,000 per *μ*L, *P* = 0.08). Also, total serum levels of TGF-*β*1 and VEGF per platelet did not differ between lung cancer cases and controls (*P* = 0.45 and 0.33, respectively).

Although kidney cancer cases did not differ from controls in total serum TGF-*β*1 or VEGF, they had lower residual levels of TGF-*β*1 (*P* = 0.02, Table2). For all analyses of TGF-*β*1 and VEGF related to cancer risk, results were similar after adjusting for sex, age at transplantation, and sirolimus use (not shown). Also, in an analysis restricted to whites, TGF-*β*1, and VEGF levels remained unassociated with NMSC (not shown).

## Discussion

In this case–control study among liver recipients, lung cancer cases had higher serum levels of TGF-*β*1 and VEGF than recipients without cancer. Notably, these elevations were present an average of 6.8 years before the development of lung cancer. These findings extend upon limited prior studies in nontransplant populations, which also found elevated levels of TGF-*β*1 for lung cancer cases 10,14. We did not see elevated serum levels of TGF-*β*1 or VEGF in recipients with other cancers, although some associations have been observed in nontransplant study populations 13,15,16,18.

Notably, several considerations point away from an etiologic interpretation of our lung cancer findings. We believe that these considerations are important because they are also relevant for interpreting results from other studies of these biomarkers. A major challenge in interpreting serum levels of TGF-*β*1 and VEGF is that they imperfectly reflect the levels at body sites where cancers develop (e.g., in this study: lung, kidney, colon, or skin). Circulating molecules derive from tissues throughout the body, so changes at one site as a cancer develops might be too small to be detectable in the circulation, or circulating levels may reflect processes elsewhere that are unrelated to cancer.

Moreover, as we came to appreciate in interpreting our results, serum levels of TGF-*β*1 and VEGF largely reflect previous platelet stores 19–21. Because of platelet degranulation that occurs during creation of a serum specimen, serum levels of TGF-*β*1 and VEGF are substantially higher than measured in plasma 19–21. Reflecting this phenomenon, and as seen previously 16,19,22,23, we observed correlations of TGF-*β*1 and VEGF with PF4 and platelet counts. Because it is unclear whether the platelet-derived fraction is relevant for cancer biology, we developed a statistical model to assess both the component of TGF-*β*1 and VEGF associated with platelet degranulation (captured by PF4) and the remaining component unrelated to platelets (estimated using the residuals from the linear regression models of TGF-*β*1 and VEGF on PF4).

Based on our statistical model, lung cancer was associated with PF4, but not with residual levels of TGF-*β*1 and VEGF, indicating that it was the platelet-related fractions of TGF-*β*1 and VEGF that were increased. Furthermore, the amount of TGF-*β*1 or VEGF in serum, when expressed as a ratio to the platelet count, did not differ between lung cancer cases and controls. Instead, platelet counts tended to be higher in lung cancer cases than controls, as observed previously 24. Thus, the most likely explanation for the higher levels of TGF-*β*1 and VEGF in lung cancer cases may be their elevated platelet counts, rather than higher circulating levels of these proteins in vivo or a higher concentration within platelets. If that is the explanation, then an etiologic contribution of TGF-*β*1 and VEGF to the development of lung cancer would not seem to be strongly supported.

Our results do not provide evidence for a role for TGF-*β*1 or VEGF in the other cancers that we studied among liver recipients. Kidney cancer was associated with lower residual levels of TGF-*β*1. However, this may be a chance finding, and we note that total serum TGF-*β*1 levels were similar in kidney cancer cases and controls. We did not see associations with NMSC, colorectal cancer, or melanoma.

Strengths of our study include its use of prediagnostic samples, which allowed measurement of TGF-*β*1 and VEGF before development of cancer. The major limitation arises from difficulties in interpreting serum levels of these proteins. To address this issue, we carefully assessed PF4 as a measure of platelet degranulation, and we examined other similar measures such as platelet counts and the serum amount of TGF-*β*1 and VEGF expressed as a ratio of the platelet count. While our approach is not definitive, it highlights the important but underappreciated effect of platelet degranulation on serum measurements. Also, while cases and controls were well matched, we had a small number of melanoma cases and, to a lesser extent, other non-skin malignancies. An additional limitation is that we lacked data on tobacco use. Prior studies that demonstrated increased plasma levels of TGF-*β*1 in lung cancer patients also did not adjust for smoking 10,14.

In conclusion, our study does not support a role for TGF-*β*1 and VEGF in causing cancer among transplant recipients treated with CNIs. We found increased levels of TGF-*β*1 and VEGF in lung cancer cases, but this was likely caused by elevations in platelet counts and platelet degranulation. The challenges that we describe related to platelet degranulation complicate interpretation of both our results and previously published studies. In future work, assessment of plasma instead of serum would probably help mitigate these issues 19,20. Researchers may also wish to measure platelet counts and PF4, to ensure that they can assess and control for platelet degranulation using statistical models similar to ours. Studies evaluating TGF-*β*1 or VEGF pathways in tumor tissues or precursor lesions may be valuable.
